# Imbalance between Pro and Anti-Oxidant Mechanisms in Perivascular Adipose Tissue Aggravates Long-Term High-Fat Diet-Derived Endothelial Dysfunction

**DOI:** 10.1371/journal.pone.0095312

**Published:** 2014-04-23

**Authors:** Marta Gil-Ortega, Luis Condezo-Hoyos, Concha F. García-Prieto, Silvia M. Arribas, M. Carmen González, Isabel Aranguez, Mariano Ruiz-Gayo, Beatriz Somoza, María S. Fernández-Alfonso

**Affiliations:** 1 Departamento de Ciencias Farmacéuticas y de la Salud, Facultad de Farmacia, Universidad CEU-San Pablo, Madrid, Spain; 2 Departamento de Fisiología, Facultad de Medicina, Universidad Autónoma, Madrid, Spain; 3 Instituto Pluridisciplinar y Departamento de Farmacología, Facultad de Farmacia, Universidad Complutense, Madrid, Spain; 4 Departamento de Bioquímica, Facultad de Farmacia, Universidad Complutense, Madrid, Spain; The Chinese University of Hong Kong, Hong Kong

## Abstract

**Background:**

The hypothesis of this study is that long-term high-fat diets (HFD) induce perivascular adipose tissue (PVAT) dysfunction characterized by a redox imbalance, which might contribute to aggravate endothelial dysfunction in obesity.

**Methods and Results:**

C57BL/6J mice were fed either control or HFD (45% kcal from fat) for 32 weeks. Body weight, lumbar and mesenteric adipose tissue weights were significantly higher in HFD animals compared to controls. The anticontractile effect of PVAT in mesenteric arteries (MA) was lost after 32 week HFD and mesenteric endothelial-dependent relaxation was significantly impaired in presence of PVAT in HFD mice (E_max_ = 71.0±5.1 *vs* E_max_ = 58.5±4.2, p<0.001). The inhibitory effect of L-NAME on Ach-induced relaxation was less intense in the HFD group compared with controls suggesting a reduction of endothelial NO availability. Expression of eNOS and NO bioavailability were reduced in MA and almost undetectable in mesenteric PVAT of the HFD group. Superoxide levels and NOX activity were higher in PVAT of HFD mice. Apocynin only reduced contractile responses to NA in HFD animals. Expression of ec-SOD and total SOD activity were significantly reduced in PVAT of HFD mice. No changes were observed in Mn-SOD, Cu/Zn-SOD or catalase. The ratio [GSSG]/([GSH]+[GSSG]) was 2-fold higher in the mesenteric PVAT from HFD animals compared to controls.

**Conclusions:**

We suggest that the imbalance between pro-oxidant (NOX, superoxide anions, hydrogen peroxide) and anti-oxidant (eNOS, NO, ecSOD, GSSG) mechanisms in PVAT after long-term HFD might contribute to the aggravation of endothelial dysfunction.

## Introduction

Perivascular adipose tissue (PVAT) is now considered a highly active endocrine organ that releases a variety of adipokines, inflammatory cytokines, and other factors which influence vascular tone in a paracrine way [1], [2]. Under physiological conditions PVAT releases a number of vasoactive substances, such as adipocyte-derived relaxing factor (ADRF) [3], [4], [5], [6], adiponectin [6], angiotensin-(1–7) [7], hydrogen peroxide (H_2_O_2_) [8], leptin [9], and nitric oxide (NO) [10], that elicit a net beneficial anti-contractile effect on vascular function and are essential for the maintenance of vascular resistance [1], [2], [8].

The amount of PVAT can vary under different physiopathological conditions. Thus, its reduction in spontaneously hypertensive rats [5], [9], [11] or in lipoatrophic mice [12] has been shown to correlate with a reduction in the production of vasodilator adipokines and also with an increase in contractile responses [5], [9], [11] and blood pressure [12]. To the contrary, a moderate enlargement of PVAT is associated with an increase of vasodilator adipokines [10]. In this context, we have shown that a short-term HFD triggers NO overproduction in PVAT contributing to the improvement of mesenteric vasodilator responses [10].

However, changes in PVAT quantity are not solely responsible for changes in vascular function. In established obesity, there might be also changes in the expression pattern of adipokines and other PVAT-derived factors, which shift the paracrine influence from a net anti-contractile effect to a pro-oxidant, pro-inflammatory and contractile environment [2]. In this context, Gao et al [13] demonstrated in a pharmacological model of obesity a loss of the anti-contractile effect of periaortic adipose tissue. New Zealand obese mice, which exhibit polygenic obesity associated to most symptoms of the metabolic syndrome, show a poor anti-contractile effect of mesenteric PVAT [6] together with increased NADPH oxidase activity and superoxide production (O_2_^-^) [14]. Similarly, short-term very HFD (60% cal from fat) has been shown to enhance both O_2_^-^ and H_2_O_2_ levels, as well as Nfc2 expression in periaortic adipose tissue [15]. In obese patients, a loss of the dilator effect of PVAT together with an increase in adipocyte area and higher expression of inflammatory markers has been reported [16].

In any case, most of the precedent findings have been identified after short-term HFD treatments and on periaortic adipose tissue, which exhibits typical characteristics of brown adipose tissue in contrast to the white phenotype of mesenteric PVAT [11]. Moreover, most studies focus on the up-regulation of pro-oxidant mechanisms (production of O_2_^-^ and H_2_O_2_, increase of NADPH oxidase activity, etc) but do not deal with eventual alterations in PVAT anti-oxidant mechanisms. Because redox alterations result from the imbalance between pro- and anti-oxidant mechanisms, our hypothesis is that long-term HFD induces a mesenteric PVAT dysfunction, which is characterized by a down-regulation of antioxidant together with the up-regulation of pro-oxidative mechanisms and leads to a loss of PVAT anticontractile effect aggravating endothelial dysfunction in resistance arteries. We have determined in mesenteric PVAT from mice receiving a HFD during 32-week: i) nitric oxide (NO) and superoxide anion (O_2_^-^) availability, ii) eNOS expression, iii) NADPH oxidase (NOX) and total superoxide dismutase (SOD) activities, iv) Cu/Zn-SOD, Mn-SOD and ec-SOD isoforms, v) catalase and levels of reduced glutathione, and vi) the role of PVAT on vascular function.

## Materials and Methods

### Animals and dietary treatment

Four-week old male C57BL/6J mice (Harlan, Spain) weighing 16–18 g were housed under controlled light (12-hour light/dark cycles from 8:00 am to 8:00 pm) and temperature (22–24°C) conditions with standard food and water *ad libitum*. After one week, animals with similar average body weight, were divided into two groups, housed 8–10 per cage, and assigned either to a control or to a high-fat diet (HFD). Control (D12450B, 10 kcal % fat, 70 kcal % carbohydrates and 20 kcal % protein; 3.85 kcal/g) and HFD (D12451, 45 kcal % fat, 35 kcal % carbohydrates, 20 kcal % protein; 4.73 kcal/g) diets were supplied by Test Diet Limited BCM IPS Ltd (London, UK). HFD and their respective control mice had free access to food during 32±1 weeks. On the last day, mice were weighed and systolic (SBP) and diastolic (DBP) blood pressure were determined through a cannula inserted in the right carotid artery connected to a pressure transducer (Statham, Harvard Apparatus GmbH, Germany). All surgery was performed under anaesthesia (80 mg·kg-1 ketamine hydrochloride and 12 mg·kg-1 xylazine hydrochloride) and all efforts were made to minimize suffering. SBP and DBP were recorded in a PowerLab system (ADInstruments). The mesenteric bed was immediately dissected. In addition, mesenteric and lumbar adipose tissue were weighed and normalized by tibia length. Blood was collected in chilled EDTA-coated polypropylene tubes in the morning. 24-hour fasting was avoided since fasting stimulates lipid mobilization, lipolysis and a reduction of mesenteric perivascular adipose tissue amount [5]. Biochemical values represent therefore postprandial concentrations. Plasma samples were frozen for biochemical determinations and isolated mesenteric arteries were used for confocal imaging studies and were also frozen at −80°C for western blot analysis. The investigation conforms to the Guide for the Care and Use of Laboratory Animals published by the US National Institute of Health (NIH publication No. 85-23, revised 1996) and was approved by the university ethics review board (Comité de Experimentación Animal de la Universidad Complutense de Madrid CEA-24-10-2008).

### Adipocyte diameter determination by confocal microscopy

Adipocyte diameter was assessed in fresh adipose tissue by confocal microscopy based on adipocyte autofluorescence. The size of the fat cell was measured by direct microscopic determination, and the mean adipocyte diameter was calculated from measurements of 100 cells per animal [5].

### Vascular reactivity in the perfused mesenteric vascular bed and in isolated mesenteric arteries cleaned of PVAT

Vascular reactivity experiments were performed as previously described [10]. Briefly, MB was perfused at a constant flow (1.5 ml min^−1^) with oxygenated (95% O_2_/5% CO_2_) Krebs-Henseleit solution (KHS, in mM, 115 NaCl, 4.6 KCl, 2.5 CaCl_2_, 25 NaHCO_3_, 1.2 KH_2_PO_4_, 1.2 MgSO_4_, 0.01 EDTA and 11.1 glucose). After equilibration (40 min), vascular contractility was assessed with 75 mM KCl. Contractile responses were determined with noradrenaline (NA, 0.1–10 µM). Relaxant responses to acetylcholine (Ach, 1 nM–0.1 mM) and sodium nitroprusside (SNP, 0.001 nM–10 µM) were analysed in vessels pre-contracted with NA (10 µM), which elicited a submaximal contraction of 90% Emax. Although 0.1 mM NA elicited maximal contraction we did not add this concentration since the bed desensitized. The nitric oxide synthase inhibitor, N_G_-nitro-L-arginine methyl ester (L-NAME, 0.1 mM), the radical scavenger, apocynin (0.1 mM) and the catalase inhibitor, 3-amino-1,2,4-triazole (3-AT, 20 mM) were pre-incubated for 20 min.

Isolated mesenteric arteries were mounted on 25 µm wires in a wire myograph (Danish Myotech, Aarhus, Denmark). After 30 min incubation, vessel wall tension and diameter were normalized following a standardized method described by Mulvany and Halpern [17]. After equilibration, vascular contractility was assessed with 75 mM KCl. Contractile responses were determined with NA (1 nM–10 µM). Relaxant responses to Ach (1 nM–0.1 mM) were analysed in MA, pre-contracted with NA (1 µM–3 µM) which elicited a submaximal contraction of 90% Emax. L-NAME (0.1 mM), apocynin (0.1 mM) and 3-AT (20 mM) were pre-incubated for 20 min.

### Determination by confocal microscopy of basal NO and O_2_^.-^ availability

NO availability was determined with the fluorescent NO indicator 4,5-diaminofluorescein diacetate (DAF-2DA) [18]. Adipose tissue-free first-order MA and PVAT were stabilized in Krebs solution (30 min, 37°C), then stained with DAF-2DA (10 µM), fixed in 4% paraformaldehyde (PFA) and mounted intact (3 mm length segments) in a small well as previously described [18]. Negative and positive controls were incubated with either L-NAME (0.1 mM) or superoxide dismutase (SOD, 15 U/ml), respectively. Under our conditions, PFA fixation did not affect DAF-2DA fluorescence [18]. Confocal projections of the MA and PVAT were then obtained and intensity levels quantified with Metamorph image analysis software (Universal Imaging Corporation, Buckinghamshire, UK).

Basal O_2_^-^ availability in MA and PVAT was determined with dihidroethidium (DHE, 3 µM) [18]. DHE permeates the cell and is directly oxidized by superoxide anion resulting in the generation of ethidium bromide, that combined with DNA is trapped in the nucleus and emits an intense red fluorescence. MA segments and PVAT were treated and analysed as previously described for DAF-2 DA protocol. Negative controls were incubated throughout the experimental period with SOD (15 U/ml).

### NADPH oxidase (NOX) and total superoxide dismutase (SOD) activities

NOX activity was measured in first-order MA segments and mesenteric PVAT by using the lucigenin-enhanced chemiluminescence method [18]. Total SOD activity was also determined in these tissues by a modified nitroblue tetrazolium (NBT)-based spectrometric assay [19] that includes bathocuproine sulfonate (BCS) as chelator agent and inhibitor of mitochondrial electron transport chain, and xanthine oxidase [19]. Briefly, 126 µl BCS/NBT mixture (1 ml BCS 0.13 mM+34.5 µl NBT 2.6 mM) were mixed with 20 µl MA or PVAT homogenate, 20 µl buttermilk xanthine oxidase (0.126 U/ml) and 20 µl potassium phosphate buffer (50 mM, pH 7.5). The mixture was incubated at 37°C and 14 µl xanthine (2.1 mM) were added. The formazan formation reaction was monitored for 20 min at 630 nm by using a microplate reader programmed in kinetic mode. The absorbance change rate (S) was calculated as the slope of the absorbance *vs* the reaction time, by using a linear regression analysis. SOD activity was expressed in U/mg protein, which was estimated from the SOD standard curve (0–6 U/ml) obtained as the S_0_/S ratio *vs* the enzyme concentration, being S_0_ the slope of the uninhibited (without SOD) reaction.

### Western blot of endothelial (eNOS), SOD isoforms and catalase (CAT)

Amounts of Cu/Zn-SOD, Mn-SOD, ec-SOD and CAT, eNOS and phosphorylated eNOS (p-eNOS) were quantified in adipose tissue-free MA and PVAT. Western blotting was performed as previously described [20]. Briefly, 30 µg protein samples were separated by 10% (eNOS and p-eNOS) or 15% (Cu/Zn-SOD, Mn-SOD, ec-SOD and CAT) SDS-PAGE gels. Primary antibodies against e-NOS (BD Transduction Laboratories, Lexington, UK; 1∶1000 final dilution), p-eNOS (Ser-1177) (Cell Signalling Technology, USA; 1∶500 final dilution), Cu/Zn-SOD (Santa Cruz Biotechnology, Germany; 1∶2000 final dilution), Mn-SOD (Santa Cruz Biotechnology, Germany; 1∶2000 final dilution), ec-SOD (StressGen; USA; 1∶1000 final dilution) and CAT (Sigma-Aldrich, Spain; 1∶2000 final dilution) were applied overnight at 4°C. After washing, appropriate secondary antibodies (anti-rabbit or anti-mouse IgG-peroxidase conjugated) were applied for 1 h. Blots were washed, incubated in commercial enhanced chemiluminiscence reagents (ECL, Amersham Bioscience, UK) and exposed to autoradiographic film. To prove equal loadings of samples, blots were re-incubated with β-actin antibody (Affinity Bioreagents, USA). Films were scanned using a GS-800 Calibrated Densitometer (Bio-Rad, Spain) and blots were quantified using Quantity One software (Bio-Rad, Spain). Expression values of peNOS were normalized with eNOS and expression values of Cu/Zn-SOD, Mn-SOD, ec-SOD and CAT were normalized with β-actin, to account for variations in gel loading.

### Chemicals

Ach was dissolved in saline. NA and SNP were dissolved in 0.01% ascorbic acid/saline. L-NAME, 3-AT and apocynin were dissolved in distilled water. DHE was dissolved in dimethylsulfoxide (DMSO) and was kept in dark conditions under argon. Ach, SNP, L-NAME, 3-AT, apocynin, DAF-2DA and DHE were obtained from Sigma Aldrich.

### Statistical analysis

All values are given as mean ± SEM. Statistical significance was analysed by one-way ANOVA or two-way ANOVA followed by Newman-Keuls post-hoc test. Statistical significance was set at p<0.05. *n* represents the number of data. Contractions are expressed in mm Hg in the perfused MB, and in mN in isolated mesenteric arteries. Relaxations are expressed as the percentage of a previous NA contraction. The maximum response (E_max_) and the negative log of the concentration producing 50% of the maximum response (pD_2_) were calculated by a non-linear regression analysis of each individual concentration-response curve. Area under the concentration-response curves (AUCs) were calculated from the individual concentration-response curve plots.

## Results

### Physiological variables after 32 week HFD

Body weight (BW), lumbar and mesenteric adipose tissue weights were significantly higher in HFD animals compared to controls (Table 1). Mesenteric adipocyte diameter was significantly higher in HFD compared to control. HFD induced an increase in leptin, insulin and glucose plasma concentrations, together with a reduction in adiponectin plasma levels (Table 1). Both systolic and diastolic blood pressure levels were similar between groups.

**Table 1:** Effect of dietary treatment on body weight, adiposity and plasma parameters.

	Control diet	High-fat diet
**Body Weight (g)**	31.4±0.9	46.4±0.7 ***
**Tibial length (mm)**	20.3±0.1	20.3±0.2
**SBP (mm Hg)**	85.7±6.3	90.1±5.5
**DBP (mm Hg)**	63.8±5.1	60.6±2.4
**Lumbar adipose tissue (mg/mm)**	14.1±1.5	36.2±4.0 *
**Mesenteric adipose tissue (mg/mm)**	19.1±2.7	44.8±3.6 ***
**Adipocyte diameter (µm)**	62.33±2.3	113.15±10***
**Leptin (ng/ml)**	13.6±2.0	28.4±6.1 *
**Adiponectin (µg/ml)**	9.9±0.4	7.8±0.4 ***
**Insulin (µg/ml)**	2.1±0.3	6.1±0.5 ***
**Glucose (mg/dl)**	191.5±28.4	236.9±25.3 *
**Triglycerides (mg/dl)**	79.6±3.6	81.5±3.0

Adipose tissue weights were normalized by using tibia length as reference. Values are mean ± S.E.M.; n = 16 determinations/group. *p<0.05, ***p<0.001, compared to their corresponding matched control groups. Newman Keuls' test.

### Anticontractile effect of PVAT is lost after 32 week HFD

**Figure 1:**
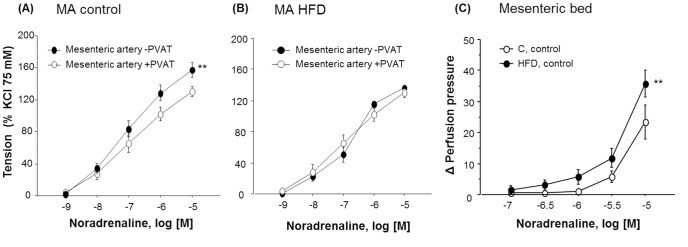
Effect of PVAT on contractile responses to noradrenaline. Cumulative concentration-response curves to noradrenaline (NA, 1 nM–10 µM) in MA with (+) and without (−) PVAT from control (C) [A] and high fat diet (HFD) animals [B]. Data are means ± S.E.M. (n≥5 animals per group). **p<0.01, compared to MA (-) PVAT. Cumulative concentration-response curves to NA (0.1–10 µM) in MB from C and HFD animals [C]. Data are means ± S.E.M. (n≥5 animals per group). **p<0.01, compared with C.

In the mesenteric bed (MB), basal perfusion pressure (control = 14.0±2.2 mm Hg; HFD = 14.7±1.6 mm Hg) and contraction to 75 mM KCl (control = 44.6±10.6 mm Hg; HFD = 38.8±6.8 mm Hg) were similar between groups. In control mice, vasoconstriction to noradrenaline (NA, 1 nM–10 µM) was smaller in mesenteric arteries (MA) with PVAT than in cleaned arteries (Figure 1A). On the contrary, the presence of PVAT did not modify contraction to NA in MA of HFD mice (Figure 1B). In the MB, contractions to NA (0.1–10 µM) were significantly more intense in HFD than in the control group (Figure 1C; one-way ANOVA, F_(1,50)_ = 8.742, p<0.01).

### Mesenteric endothelial dysfunction is aggravated in presence of PVAT after 32 week HFD

Basal perfusion pressure (C = 14.0±2.2 mm Hg; HFD = 14.7±1.6 mm Hg) and contractions to 75 mM KCl (C = 44.6±10.6 mm Hg; HFD = 38.8±6.8 mm Hg) were similar between control and HFD groups suggesting no mechanic interference of PVAT amount in the HFD group.

**Figure 2:**
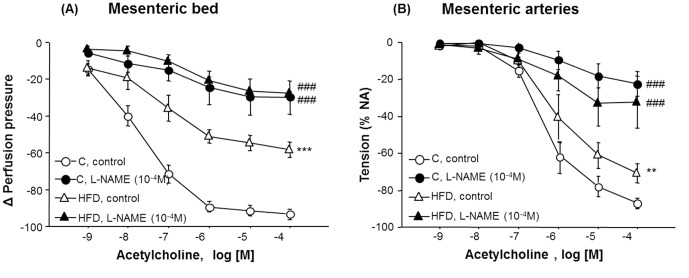
Contribution of endothelial nitric oxide to Ach-mediated relaxant responses. Cumulative concentration-response curves to acetylcholine (Ach, 1 nM–0.1 mM) in MB [A] and in MA [B] from control (C) and high fat diet (HFD) animals in absence/presence of L-NAME (0.1 mM). Data are means ± S.E.M. (n≥5 animals per group). ***p<0.001;**p<0.01, compared to C; ^###^p<0.001 compared to their corresponding matched control groups.

Relaxation to Ach (1 nM–0.1 mM) was impaired in the MB of the HFD group (Figure 2A, Table 2; one-way ANOVA, F_(1,60)_ = 113.1, p<0.001). In presence of the NOS inhibitor, L-NAME (0.1 mM), relaxations to Ach were similar between groups (Figure 2A, Table 2). However, the inhibitory effect of L-NAME on Ach-induced relaxation was less intense in the HFD group compared with controls (two-way ANOVA, F_(1,84)_ = 18.5, p<0.001), suggesting a reduction of endothelial NO availability in HFD. Indomethacin (3 µM) or indomethacin (3 µM) plus L-NAME (0.1 mM) similarly affected control and HFD MB reactivity to Ach, demonstrating that the contribution of prostanoids and EDHF to Ach-induced relaxation were not modified by HFD (results not shown). Moreover, relaxation to SNP (0.001 nM–10 µM) was similar in both groups (E_max Control_ = 90.5±3.7%; E_max HFD_ = 90.6±2.0%), indicating that the activity/sensitivity of the soluble guanilylcyclase is not modified by long-term HFD.

**Table 2:** E_max_ and pD_2_ values of Ach-induced relaxation in mesenteric arteries (MA-PVAT) and in the perfused mesenteric bed (MB).

	Control diet			High-fat diet		
	E_max_	pD_2_	AUC	E_max_	pD_2_	AUC
Ach (MA)	87.0±3.1	6.1±0.1	200.6±17.2	71.0±5.1^#^	5.8±0.2	149.7±23.9
Ach+L-NAME (MA)	20.5±9.2***	5.7±0.4	42.1±26.1**	30.7±15.6***	5.7±0.4	87.5±44.6
Ach (MB)	93.6±2.8	7.6±0.2	346.6±11.5	58.5±4.2^###^	7.1±0.3	197.2±22.8^###^
Ach+L-NAME (MB)	31.2±9.1***	6.9±0.7	98.5±31.3***	22.9±0.9^###^	6.6±0.3	78.3±7.5^***^

MA (Isolated mesenteric arteries), MB (perfused mesenteric bed). E_max_, is the maximal relaxation to acetylcholine in % of precontraction to noradrenaline. pD_2_, is the negative logarithm of molar concentration of Ach causing half maximal responses. AUC, is the area under concentration-response curves expressed in mmHg for MB and mg for MA. Data are expressed as mean ± S.E.M., n≥5. **p<0.01, ***p<0.001, compared to their corresponding matched control groups. ^#^p<0.05, ^###^p<0.001 compared to the control group.

Relaxation to Ach in MA was also significantly reduced in HFD animals (Figure 2B, table 2, one-way ANOVA, F_(1,48)_ = 10.5, p<0.01). However, the impairment of relaxation was significantly higher in the MB than in cleaned MA (20%, two-way ANOVA, F_(1,108)_ = 19.5, p<0.001), suggesting an important contribution of PVAT to endothelial dysfunction.

Preincubation with L-NAME significantly reduced relaxation to Ach in MA of both groups (one-way ANOVA, F_(1,30)_ = 3.5; p<0.001), although inhibition in the HFD group was lower compared to controls (two-way ANOVA, F_(1,96)_ = 11.7, p<0.001). Concentration-response curves to SNP (0.001 nM–10 µM) were similar between groups (results not shown) excluding changes in smooth muscle sensitivity.

### Endothelial NO availability is reduced due to an increase in superoxide anion levels in isolated MA

**Figure 3:**
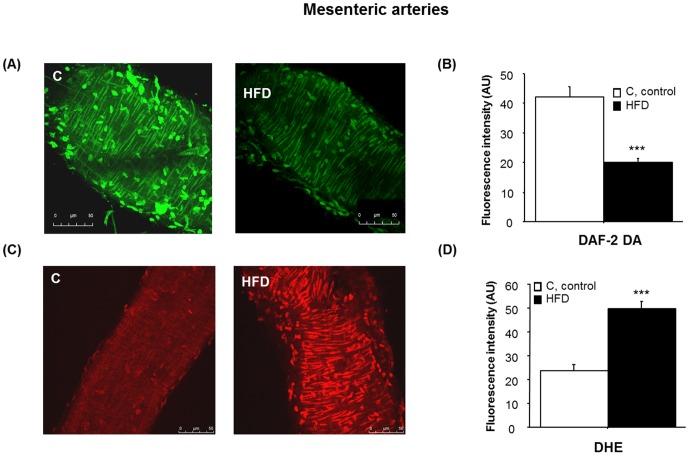
Nitric oxide and superoxide anion availability in mesenteric arteries. [A] Confocal projections showing *in situ* NO generation, determined with DAF2-DA (10^−5^ M), in MA. Adventitial cells are round; vascular smooth muscle cells are elongated and perpendicular to blood flow; endothelial cells are elongated and parallel to blood blow. [B] Fluorescence intensity in MA from control (C) and high fat diet (HFD) animals. Data are means ± S.E.M. (n≥5 animals per group). ***p<0.001 compared to C. [C] Confocal projections showing *in situ* superoxide generation determined with dihydroethidium (DHE, 3 µM) in MA and [D] quantification of DHE fluorescence intensity in MA from C and HFD. Data are means ± S.E.M. (n = 5 animals per group). ***p<0.001 compared to C.

In order to confirm changes in endothelial NO availability suggested by vascular reactivity studies, basal endothelial NO availability in MA devoid of PVAT was determined with the fluorescent NO indicator, DAF-2DA. As expected, fluorescence intensity emitted by MA was significantly lower in HFD animals than in controls (Figure 3A and 3B, one-way ANOVA, F_(1,19)_ = 39.6, p<0.001). To the contrary, basal level of O_2_^-^ determined by quantifying DHE fluorescence intensity (Figure 3C and D), was more than 2-fold higher in MA from the HFD group (one-way ANOVA, F_(1,29)_ = 29.5; p<0.001).

**Figure 4:**
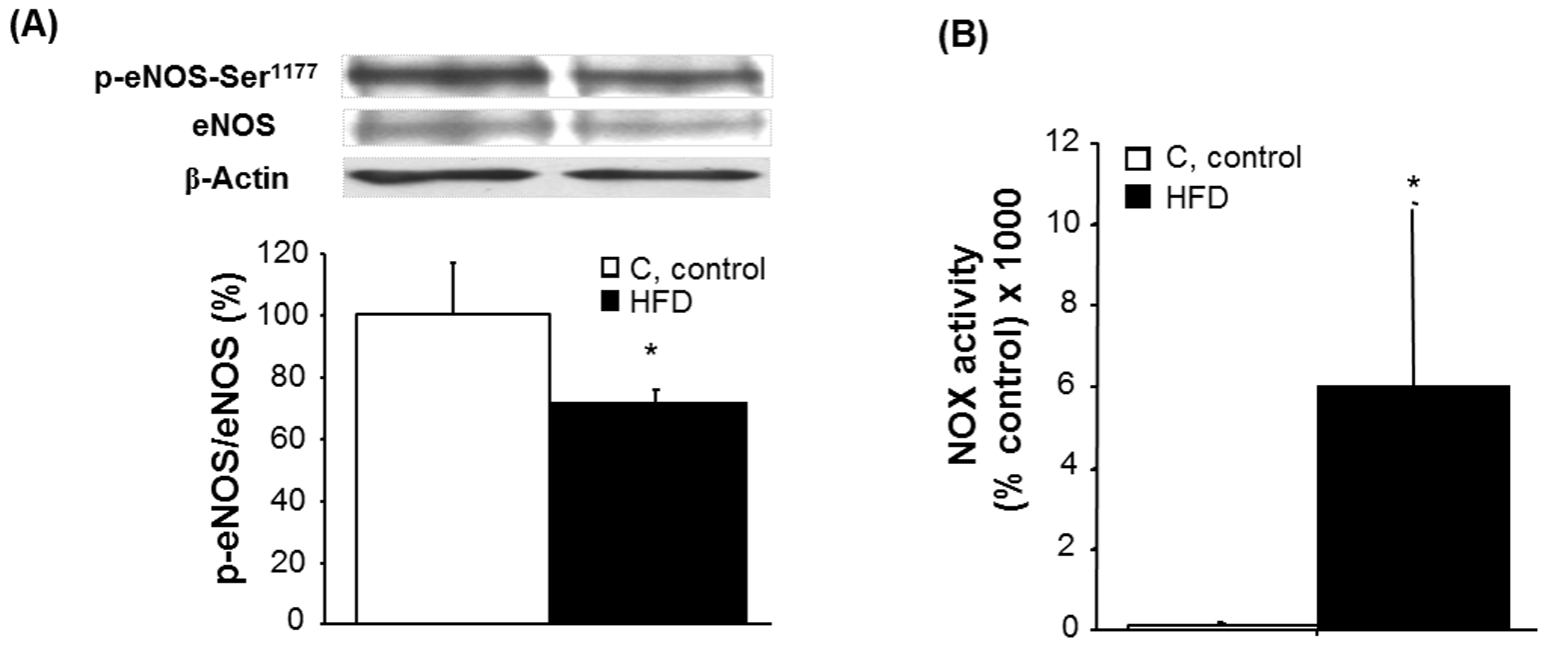
eNOS phosphorylation and NOX activity in mesenteric arteries. [A] Representative immunoblots of p-eNOS in MA. Diagram bars show the result of densitometric analysis of p-eNOS immunoblots, expressed as percentage of p-eNOS/eNOS in the control (C) group. Data are means ± S.E.M. (n≥5 animals per group). *p<0.05 compared to C. [B] NOX activity in MA. Results are expressed as percentage of NADPH oxidase activity in C animals. Data are means ± S.E.M. (n≥5 animals per group). *p<0.05 compared to C.

Western blot revealed that basal level of phosphorylated eNOS (p-eNOS-Ser^1177^) was significantly lower in HFD animals (Figure 4A; one-way ANOVA, F_(1,14)_ = 7.8, p<0.05).

Moreover, we detected a significant increase in NOX activity in MA from HFD animals (Figure 4B) which explains the increase in O_2_^.-^. Altogether, these data demonstrate that reduction in NO availability in MAs from HFD results from both a reduced NO production together with a higher NO inactivation by the increase of oxidant species.

A negative correlation was found between DAF-2DA-induced fluorescence intensity and plasma leptin levels (p<0.01; r = 0.7; F_(1,14)_ = 13.3) together with a positive correlation between DAF-2 DA-induced fluorescence intensity and plasma adiponectin levels (p<0.001; r = 0.82; F_(1,21)_ = 43.6). Similarly, we also found a negative correlation between plasma leptin and p-eNOS (p<0.001; r = 0.93; F_(1,14)_ = 82.5), and a positive correlation between plasma adiponectin and p-eNOS (p<0.01; r = 0.74; F_(1,14)_ = 16.7). To the contrary, DHE fluorescence intensity negatively and positively correlated with adiponectin (p<0.001; r = 0.8; F_(1,21)_ = 36.2) and leptin (p<0.01; r = 0.73; F_(1,14)_ = 15.7), respectively.

### PVAT NOX activity and superoxide anion levels are increased after long-term HFD

**Figure 5:**
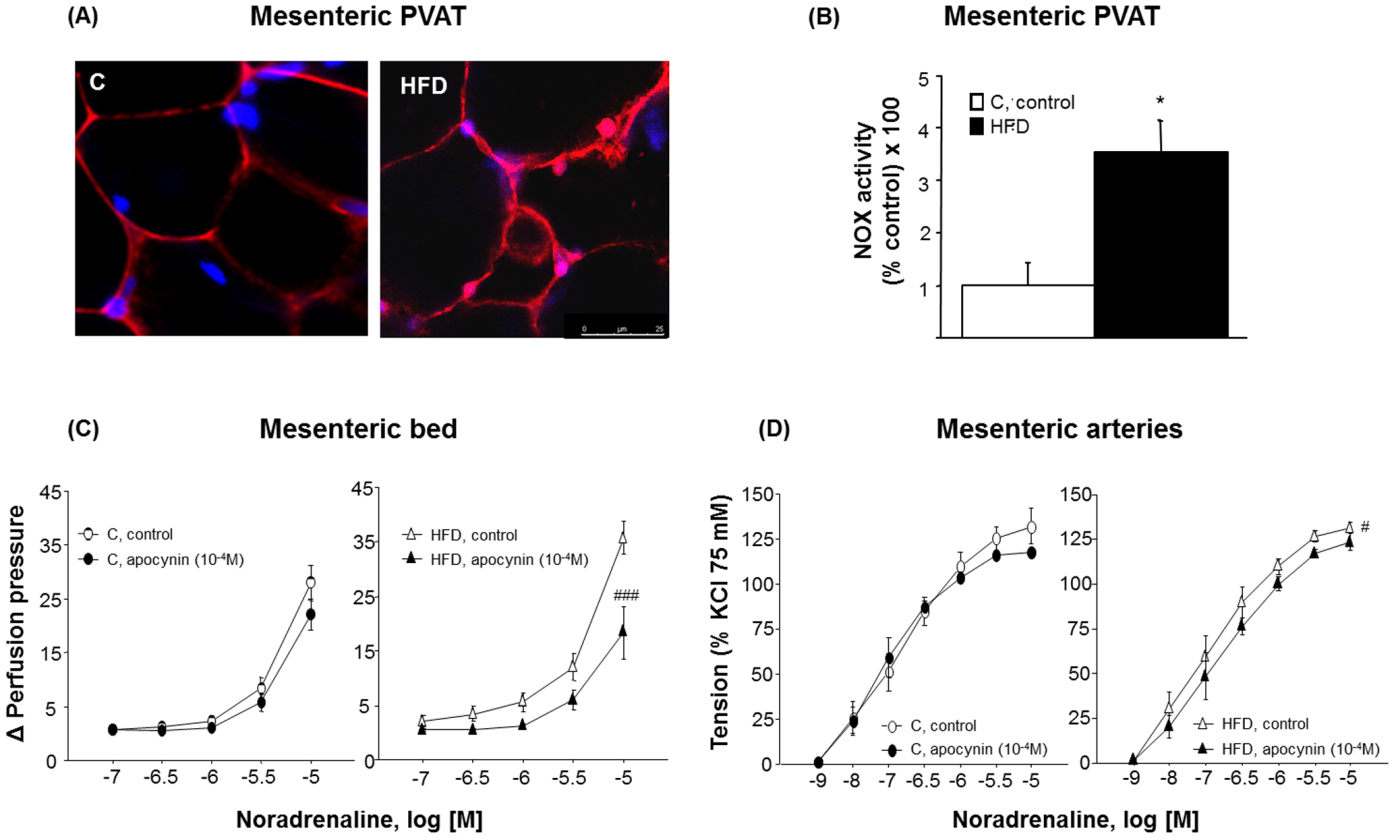
Contribution of pro-oxidant systems on contractile responses to noradrenaline in both mesenteric arteries and the mesenteric bed. [A] Confocal projections showing *in situ* superoxide generation determined with dihydroethidium (DHE, 3 µM) in mesenteric PVAT from control (C) and high fat diet (HFD) animals. [B] NOX activity in mesenteric PVAT. Results are expressed as percentage of NOX activity in C. Data are means ± S.E.M. (n≥5 animals per group). *p<0.05 compared to C animals. [C] Cumulative concentration-response curves to noradrenaline (NA, 0.1–10 µM) in MB and [D] cumulative concentration-response curves to NA (1 nM–10 µM) in MA from C and HFD animals in absence/presence of apocynin (0.1 mM). Data are means ± S.E.M. (n≥5 animals per group). ^#^p<0.05; ^###^p<0.001 compared to their corresponding matched control groups.

To better identify the mechanisms involved in the reduction of the PVAT-derived anticontractile effect previously observed in HFD animals, both pro-oxidant and anti-oxidant mechanisms were evaluated. PVAT from HFD animals exhibited a significant increase in DHE-induced fluorescence co-localizing with nuclei DAPI-staining (Figure 5A). NOX activity (Figure 5B) was 3.5-fold higher in HFD mice. Moreover, the ratio [GSSG]/([GSH]+[GSSG]) was 2-fold higher in the mesenteric PVAT from HFD animals compared to controls (C = 22.2±8.6% *vs* HFD = 55.5±6.6%; one-way ANOVA, F_(1,7)_ = 9.8, p<0.05), thus demonstrating an increased contribution of pro-oxidant systems. However, NO availability in PVAT was almost undetectable and similar between groups (C = 21.1±4.4 *vs* HFD = 20.4±1.1), as well as eNOS expression and phosphorylation (p-eNOS) (results not shown).

### Role of increased adipose oxidative stress on vascular responses

The contribution of O_2_^-^ to NA-induced responses was evaluated in absence or presence of apocynin (0.1 mM), a scavenger of radicals and direct inhibitor of ROS-induced signaling in vascular cells [21]. Contractions to NA were significantly higher in the HFD group compared to controls (one-way ANOVA, F_(1,60)_ = 8.0, p<0.01; Figure 5C and D). Apocynin only reduced the contractile responses to NA in HFD animals (two-way ANOVA, F_(1,80)_ = 4.1; p<0.05; Figure 5C and 5D). This effect was more pronounced in presence of PVAT, suggesting that the paracrine contribution of O_2_^-^ to endothelial dysfunction was enhanced in this group.

### Long-term HF diet reduces ec-SOD expression and SOD activity in PVAT

**Figure 6:**
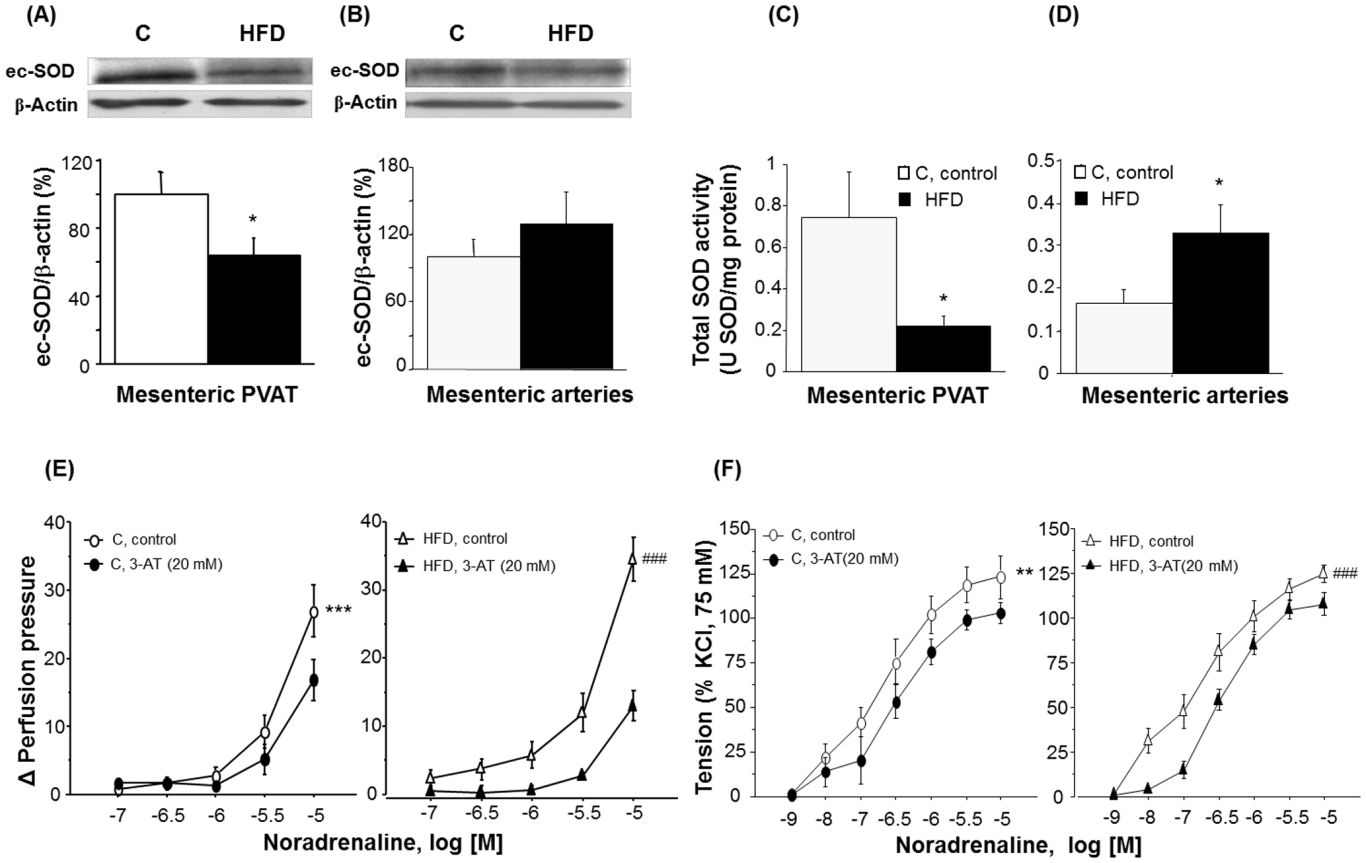
Contribution of antioxidant systems on contractile responses to noradrenaline in both mesenteric arteries and the mesenteric bed. Effect of HFD on ec-SOD protein expression in mesenteric PVAT [A] and in MA [B]. Diagram bars show the result of densitometric analysis of ec-SOD immunoblots, expressed as percentage of ec-SOD in the control (C) group. Data are means ± S.E.M. (n≥5 animals per group). Total SOD activity in mesenteric PVAT [C] and in MA [D]. Data are presented as means ± S.E.M. (n≥5 animals per group). *p<0.05 compared to C animals. [E] Cumulative concentration-response curves to noradrenaline (NA, 0.1–10 µM) in MB and [F] cumulative concentration-response curves to NA (0.1–10 µM) in MA from C and HFD animals in absence/presence of 3-amino-1,2,4-triazole (3-AT, 20 mM). Data are means ± S.E.M. (n≥5 animals per group). ^##^p<0.01; ^###^p<0.001 compared to their corresponding matched control groups.

The expression of the SOD isoforms, Cu/Zn-SOD and Mn-SOD, in mesenteric PVAT and in MA was similar between groups (results not shown). No differences were observed between groups and tissues in catalase levels (C = 100.0±10.2% *vs* HFD = 118.12±6.0%; one-way ANOVA, F_(1,12)_ = 2.4, p = 0.15). In contrast, ec-SOD expression was significantly reduced in PVAT of HFD mice (Figure 6A) with no differences in MA between groups (Figure 6B). Total SOD activity was significantly reduced in PVAT, but significantly increased in MA of HFD animals (Figures 6C and 6D).

The contribution of H_2_O_2_ to NA-induced responses was evaluated in absence or presence of the catalase inhibitor, 3-amino-1,2,4-triazole (3-AT, 20 mM), which selectively inhibits catalase activity at concentrations as high as 50 mM [22]. In the MB, the contractile response to NA (0.1–10 µM) was significantly reduced in both groups (2-ANOVA, F_(1,80)_ = 13.757, p<0.001) although it was more pronounced in the HFD group (Figure 6E). However, while the contractile response to NA (1 nM–10 µM) was significantly reduced in the MA of both groups, no differences were detected between control and HFD animals (Figure 6F).

## Discussion

In this study we show for the first time that long-term HFD induces PVAT dysfunction characterized by i) a substantial reduction in eNOS and NO production, ii) a decrement in ec-SOD expression and total SOD activity, and iii) an increase of NOX activity and superoxide anion release. Moreover, our study provides the first evidence of ec-SOD expression in PVAT as well as its different regulation between PVAT and the vascular wall in obesity. We suggest that the imbalance between antioxidant and pro-oxidant mechanisms in PVAT evoked by long-term HFD might contribute to vascular oxidative stress, thus aggravating endothelial dysfunction.

Vascular function assays, carried out in both isolated mesenteric artery (MA) and in the whole mesenteric bed (MB), suggest that PVAT elicits an anti-contractile effect that positively correlates with the amount of adipose tissue surrounding the artery (PVAT in first-order MA branches is about 4–6 mg/2 mm segment artery *vs* 372±52 mg PVAT in MB), as we had previously suggested [5], [10]. To better highlight changes in PVAT properties in HFD mice, we compared between responses in the intact MB and in first-order MA cleaned from fat. We are aware that this is a limitation of the study since functional characteristics of MA and MB are different. To validate functional results, expression studies (SODs, NOX, catalase), glutathione, NO and superoxide level determination and enzyme activity measurements (total SOD and NOX) were quantified in first-order MA with and without PVAT.

In our long-term HFD model we observed a substantial endothelial dysfunction in absence of PVAT, as previously described in experimental models of obesity [23]. The endothelial dysfunction was associated to a reduced endothelial p-eNOS and NO availability, which was independent of EDHF, prostanoids or smooth muscle sensitivity to NO, but linked to an increase in NOX activity and O_2_^-^ levels. O_2_^.-^ concentration depends on the balance between its production and dismutation rate by the various superoxide dismutases (SODs), the copper-zinc SOD (Cu/Zn-SOD), the manganese SOD (Mn-SOD), and the extracellular form of Cu/Zn-SOD (ec-SOD). In the vascular wall, total SOD activity appeared to be increased, probably aimed to compensate increased O_2_^.-^. In these context, preincubation with apocynin (10 mM) reduced contractions to NA in HFD but not in controls, suggesting that the increase in total SOD activity is insufficient to compensate NO reduction and endothelial dysfunction in HFD.

Endothelial dysfunction was aggravated in presence of PVAT, suggesting that its beneficial anti-contractile effect observed after short-term HFD [10] is lost after 32-week HFD. The deleterious influence of PVAT might be the result of i) the down-regulation of both eNOS expression and NO production (that reaches almost undetectable levels), ii) the increase in NOX activity and O_2_^-^ levels and iii) down-regulation of ec-SOD and total SOD activity. The 2-fold increase in GSSG/(GSH/GSSG) ratio detected in HFD might be aimed at compensating oxidative damage, but seems, however, to be insufficient to balance the increase in O_2_^.-^ availability and endothelial dysfunction. Changes observed in PVAT and in vascular function might be primary due to HFD or secondary to obesity. Our model cannot be strictly considered as a model of metabolic syndrome, as DIO mice did not display hypertension. Otherwise, as previously reported by ourselves, 32-wk HFD leads to insulin resistance, moderate hyperleptinemia and hepatic steatosis. Nevertheless basal levels of trigycerides and free fatty-acids were not different between controls and HFD mice [24]. However, we cannot exclude that hyperinsulinemia and/or hyperglycaemia could trigger vascular damage, thus contributing to vascular dysfunction, independently of the effect of PVAT.

The presence of ec-SOD in PVAT constitutes a novelty itself, as this enzyme had never been identified previously in PVAT. In fact ec-SOD, synthetized by fibroblast and smooth muscle cells [25], had been previously identified as a pivotal element to protect the vascular wall from O_2_^.-^, thus allowing endothelial NO to reach the vascular smooth muscle layer [26]. Reduced ec-SOD expression has been associated with pathological conditions affecting vascular function, such as atherosclerosis or coronary artery disease [27]. However, deficiency in ec-SOD does not alter baseline blood pressure [28] http://atvb.ahajournals.org/cgi/content/full/24/8/1367-R93-015610#R93-015610, despite increased vasoconstrictor responses [29], as we also can observe in our model.

**Figure 7:**
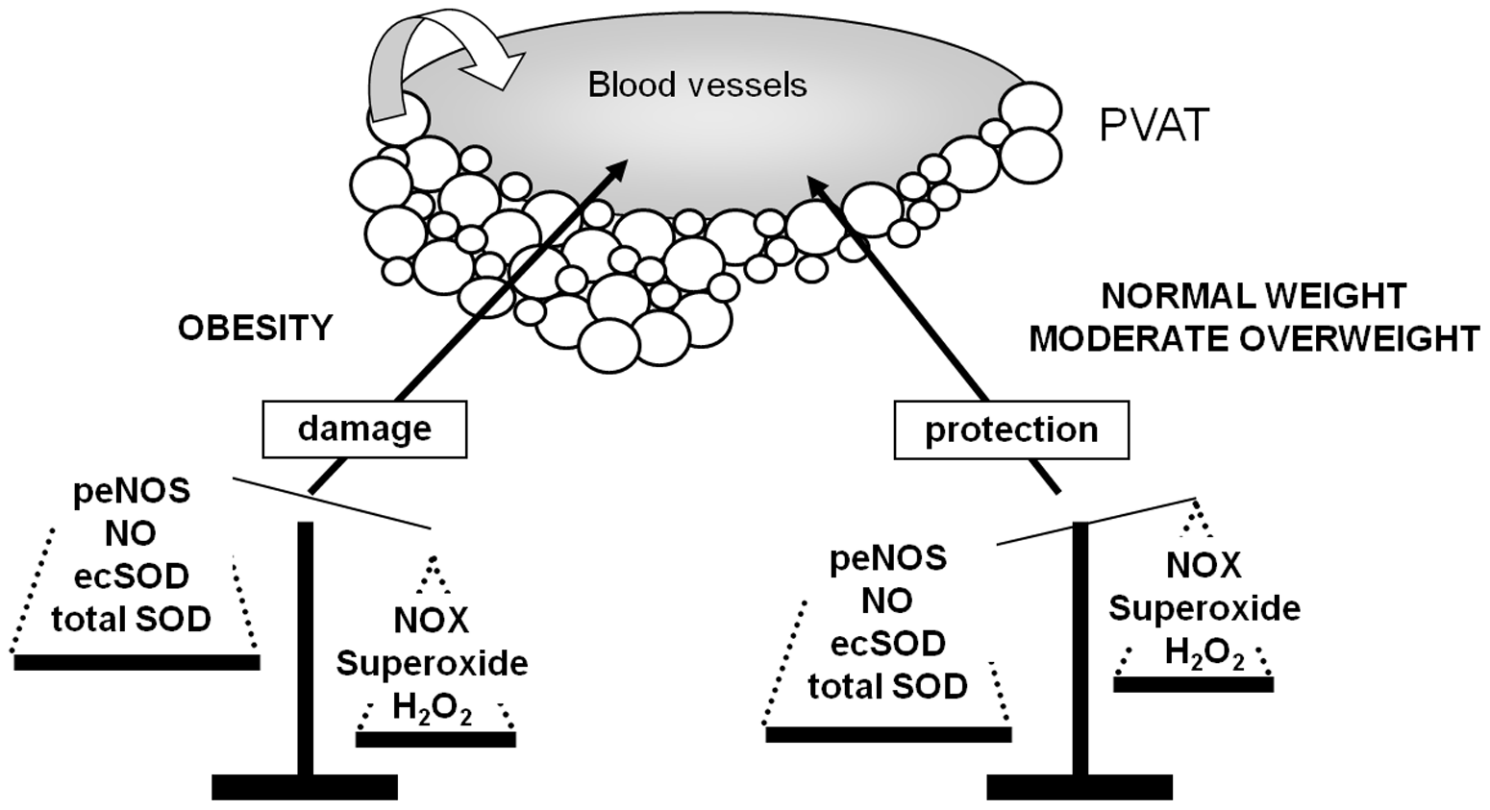
Representative diagram with a possible mechanism explaining the effect of long-term high fat diet (HFD) on endothelial dysfunction. Adipokine dysregulation in PVAT (increase in leptin release together with reduced adiponectin levels) lead to an increase in NOX activity but a reduction in total SOD activity and ec-SOD expression. Therefore, obesity induces a huge increment of superoxide levels in PVAT. Circulating and PVAT-derived adipokines might also lead to an increased NOX activity and consequently, to enhance superoxide and H_2_O_2_ levels in the vascular wall. PVAT-derived adipokines might also contribute to a reduction in eNOS phosphorylation and, consequently to reduced NO availability that accounts for endothelial dysfunction aggravated by PVAT-derived superoxide.

A pivotal question evoked by our study deals with the ability of PVAT O_2_^.-^, which displays a short half-life and low diffusion radius, to reach the media and the endothelium. We propose two possible answers. First, O_2_^.-^ is rapidily converted to H_2_O_2,_ which is cell-permeable and highly stable [30]. H_2_O_2_ is able to exert paracrine vasoactive and structural effects in the media independently of its source, i.e. adventitia, intima or PVAT [31], [32]. Here, the overall contribution of H_2_O_2_ to vascular function was higher in presence of PVAT in the HF group (Figure 6) since it is the sum of H_2_O_2_ derived from both PVAT (although lower than in the control group) and the vessel wall. Secondly, in a context of high O_2_^.-^ availability due to an increased NOX and a reduced SOD activity, as well as lack of NO, we cannot exclude the possibility of O_2_^.-^ diffusion contributing to reduce endothelial NO availability in a paracrine way (Figure 7).

A further question raised by our data concerns the eventual influence of hyperleptinemia and/or hypoadiponectinemia on vascular O_2_^-^ and H_2_O_2_ production. Leptin has been shown to increase NOX activity and O_2_^-^ production in the vascular wall as well as to promote GPx activation to remove excessive H_2_O_2_ production [33], [34]. Moreover, hypoadiponectinemia is closely associated with endothelial dysfunction in humans [35] and adiponectin knock-out mice show reduced p-eNOS levels [36]. Interestingly, the reduction of adiponectin expression in an adipocyte cell line parallels the down-regulation of ec-SOD and plasma ec-SOD levels inversely correlate with body mass index [37].

In conclusion, we provide new evidence that PVAT dysfunction after a long-term HFD contributes to a deficient management of perivascular adipose superoxide that aggravates endothelial dysfunction. Our results suggest that endothelial dysfunction triggered by HFD is aggravated by the increase of oxidative stress in PVAT, describing a role for ec-SOD in this altered redox balance. Altogether, these findings demonstrate that changes in the expression pattern of PVAT-derived anti-oxidant and pro-oxidant factors shift the paracrine influence of PVAT from a net anti-contractile effect to a pro-oxidant, pro-inflammatory and contractile environment and support a prominent influence of mesenteric PVAT in endothelial dysfunction in diet-induced obesity, as previously suggested for periaortic adipose tissue [13], [15], [16].
